# Assimilating to Hierarchical Culture: A Grounded Theory Study on Communication among Clinical Nurses

**DOI:** 10.1371/journal.pone.0156305

**Published:** 2016-06-02

**Authors:** MinYoung Kim, Seieun Oh

**Affiliations:** 1 College of Nursing, Jeju National University, Jeju-si, Jeju Special Self-Governing Province, South Korea; 2 Department of Nursing, Dankook University, Cheonan-si, Chungnam, South Korea; University of Pittsburgh, UNITED STATES

## Abstract

The purpose of this study was to generate a substantive model that accounts for the explanatory social processes of communication in which nurses were engaged in clinical settings in Korea. Grounded theory methodology was used in this study. A total of 15 clinical nurses participated in the in-depth interviews. “Assimilating to the hierarchical culture” emerged as the basic social process of communication in which the participants engaged in their work environments. To adapt to the cultures of their assigned wards, the nurses learned to be silent and engaged in their assimilation into the established hierarchy. The process of assimilation consisted of three phases based on the major goals that nurses worked to achieve: getting to know about unspoken rules, persevering within the culture, and acting as senior nurse. Seven strategies and actions utilized to achieve the major tasks emerged as subcategories, including receiving strong disapproval, learning by observing, going silent, finding out what is acceptable, minimizing distress, taking advantages as senior nurse, and taking responsibilities as senior nurse. The findings identified how the pattern of communication in nursing organizations affected the way in which nurses were assimilated into organizational culture, from individual nurses’ perspectives. In order to improve the rigid working atmosphere and culture in nursing organizations and increase members’ satisfaction with work and quality of life, managers and staff nurses need training that focuses on effective communication and encouraging peer opinion-sharing within horizontal relationships. Moreover, organization-level support should be provided to create an environment that encourages free expression.

## Introduction

Proactive and effective communication in an organization has been known as one of the critical factors that exert a major influence on work outcomes as well as individual members’ emotional health [1]. More importantly, work environments under which the members do not feel comfortable to voice their opinions or raise work-related issues could cause conflict between members, compromise work quality and subsequently negatively impact the entire organization [2].

Valuing the employee’s voice is also crucial in in any health care organization. When constructive organizational communication is hampered, its consequences are not limited to the organization’s success; patient safety and care quality, and subsequently the patients’ health, can be seriously compromised [3]. Maintaining a high-quality work environment, including creating an atmosphere that encourages nurses to voice their concerns and opinions relevant to their work, can reduce the members’ emotional distress and potential for errors; and improve patients’ safety outcomes [1, 4].

Nursing units in hospital settings are primarily shift-based work environments where continuity of patient care needs to be maintained throughout all shifts; nursing performance and care outcomes in previous shifts can influence the workload of those working during subsequent shifts. Thus, care quality and subsequent health outcomes of patients depend upon effective communication between nurses [5].

In addition to the shift-based system, more conditions leading to distress exist in work environments for nurses in Korea than for nurses in other countries. Registered nurses (RNs) in Korea have greater workloads than their counterparts in other countries. The minimum legal requirement of patient care in Korean hospitals is one RN to 13 patients in general ward settings, while comparable settings in the U.S. and Japan allow ratios of one to five and one to seven, respectively; additionally, those with 300 or less bed capacity usually do not follow this regulation, which results in greater burdens for nurses working at small hospitals [6].

In addition, medical institutions in Korea have recently been forced to engage in fierce competition to provide high-quality medical services as well as generate profit [7]. A high grade or certification from national or international institutions evaluating medical service quality, such as the National Accreditation Board for Medical Institutions or the Joint Commission International (JCI) Accreditation, is one of the indicators that a hospital has succeeded in its pursuit of these goals. Nurses are regularly the most-represented of employees in a medical setting and are often required to complete tasks related to such things as accreditation in addition to their regular nursing duties [7], which can lead to stress and emotional exhaustion that is sufficiently serious to cause them to consider resignation [8]. Under such a work atmosphere, voicing objections or raising questions can be misconstrued as a lack of concern towards the provision of high-quality service to patients.

More importantly, national cultural factors such as power hierarchies and the preservation of dignity can negatively affect official communication in an organization [9]. Specifically, a cultural environment emphasizing a nursing unit hierarchy further exacerbates the psychological burdens on clinical nurses, creating peer conflict and hindering effective and constructive communication within the institution [10]. Although findings from previous research imply that the culture of nursing in Korea can negatively affect communication at an individual as well as organizational level, there is a paucity of studies further investigating how nurses in clinical settings communicate and how it affects care quality and the nursing staff’s work-related quality of life.

To fill this gap, we conducted the current study utilizing the Grounded Theory methodology to generate a substantive model that explains the communication processes of nurses in clinical settings in Korea. Grounded Theory methodology was selected for this research due to its suitability for studying under-researched areas that require the identification of fundamental issues and related processes [11]. Findings of this research grounded on data will provide information critical to improving the effectiveness of organizational communication and provide better work environments for nurses worldwide.

## Methods

### Characteristics of the Participants

Eligible study participants included registered nurses who had three or more years of experience in clinical nursing. Three or more years was selected as the needed amount of work experience as it is the amount of time it takes for nurses to likely build confidence in their ability to perform routine care tasks and thus be able to see the broader spectrum of nursing care in their organizations. Other criteria of eligibility for participation in this study were not defined in order to enhance and refine the emerging model. Participants were recruited from general hospitals in three Korean cities, C, S, and J. A total of 15 registered nurses participated in the study. No one invited to this study refused participation. The age of the participants ranged from 27 years to 43 years, with the average being 31.6 years (SD = 4.5, median = 31). At the time of the interviews, six were working in intensive care units, seven were in general wards, and two were in special units.

All participants were female. Length of nursing experience ranged from 40 to 228 months, with the average of 90.6 months (SD = 49.9, median = 84). The average level of the participants’ education was 16.4 years. In summary, study participants tended to be highly educated and in their early thirties. Four participants (26.6%) had been employed by two or more hospitals over the course of their careers.

### Data Collection and Analysis

All interviews for this study were conducted in person at a participant-chosen time and location, including in small hospital conference rooms, the researcher’s office, or a quiet spot near a participant’s workplace. Interviewers and participants in this study were all female. No one unrelated to this study was present at any of the interviews. Interviews usually took about 90 minutes, with a range from 40 minutes to 1 hour 40 minutes. Both of the authors conducted in-depth interviews with the same interview guidelines (S1 and S2 Files). All interviews were audio-recorded with participants’ permission.

At the beginning of each interview the researcher administered an informed consent procedure that included an explanation of why this research was being conducted. After the introduction, the interviews started with a set of broad open-ended questions (S1 File): “Could you please tell me how you are doing in your work these days?”; “Tell me how communication within your workplace is going”; and “Could you tell me what are the difficulties regarding communication among your coworkers?” Interview guidelines for later interviews (S2 File) also included these three anchor questions as well as probes to elicit information about emerging codes. Theoretical or methodological memos were written whenever ideas regarding data analysis came to mind.

The initial purposive sampling was done to identify “good informants,” those who had experienced the phenomenon under study and were reflective and willing to share their experiences [12]. After the first purposive sampling, theoretical sampling was performed throughout the study to saturate previously-emerged codes and categories and to gain variation of categories in their properties and dimensions with varying conditions as much as possible [13]. Newly collected data were compared to the pre-analyzed categories in terms of similarities and differences. Transcribed data were coded independently by the two authors, and the results of analyses were discussed to reach complete agreement between the study’s authors.

### Establishing Trustworthiness of the Data

We made efforts to build trustworthiness based on criteria delineated by Lincoln and Guba [14]: credibility, transferability, dependability, and confirmability. Four methods were employed to obtain trustworthiness in research procedures and data analysis: peer debriefing, member checking, material collection for an audit trail, and thick description of the study context. The authors served as peer debriefers for each other by examining each other’s bias as a researcher and corroborating that individual analysis of data was understandable and agreeable to others [14]. Since the authors have different academic and clinical backgrounds and interests, reading and discussing each other’s interpretation of data helped identify implicit preconceptions on the data. This also helped avoid arbitrary implementation of interview procedures; in particular, reviewing how the interviewer implemented interview questions and subsequent probes and examining if there was any interviewer influence on participants’ answers were particularly helpful. Three study participants reviewed the summary of study results to provide member checking. These volunteers confirmed that the summarized findings captured the essence of their experiences. All materials related to the study data and analysis were comprehensively collected for an audit trail. Lastly, contextual information about the research findings was described with as much detail as possible so that readers can evaluate whether the findings can be transferable or not.

### Protection of Human Rights and Ethical Considerations

This study was approved by the Human Subjects Committee at the first author’s institution (#2013–18). Protection of the participants’ human rights and privacy was carefully planned ahead and executed throughout the study.

The interview location was selected by participants. At the beginning of each interview session, an informed consent procedure was performed. The interviewer (either MK or SO) explained the study’s purpose and procedures, benefits and potential risks of participation, and the participant’s right not to answer any question and to withdraw from the study at any time. Participants were allowed to ask questions and express concerns; all questions were answered before signing.

Study data were protected following established protocols: signed consent forms, demographic information sheets, and digital voice-recordings were stored separately from each other in locked cabinets. A code number was provided to each study participant and any identifiable information, e.g., names or hospital identifiers, was not linked with the digitally recorded interview to ensure confidentiality of the data. Electronic files of the audio-recorded and written interview data were stored in password-protected computer files accessible only by the authors. Participants received gift cards worth about $25 USD each for their time and contribution to nursing knowledge.

### Researcher Preparation

The first author (MK), who majored in nursing management, has ten years’ experience as a nurse specialist in intensive care and internal medicine at a general hospital, completed several courses related to qualitative study during her graduate program, and has collected data from cancer patients via in-depth interviews. The second author (SO) received a doctorate degree for a qualitative research study utilizing Grounded Theory methodology. She has conducted various qualitative studies using Grounded Theory methodology and has nine years of clinical experience in critical care nursing at a university hospital. During the entire research period, the researchers continued to hold discussions and share opinions to maintain rigor in all aspects of the research including participant recruitment, interview questions, and data analysis. The researchers also engaged in continued self-examination and discussion to prevent personal biases or experiences from influencing the interpretation of the results.

## Results

**Fig 1:**
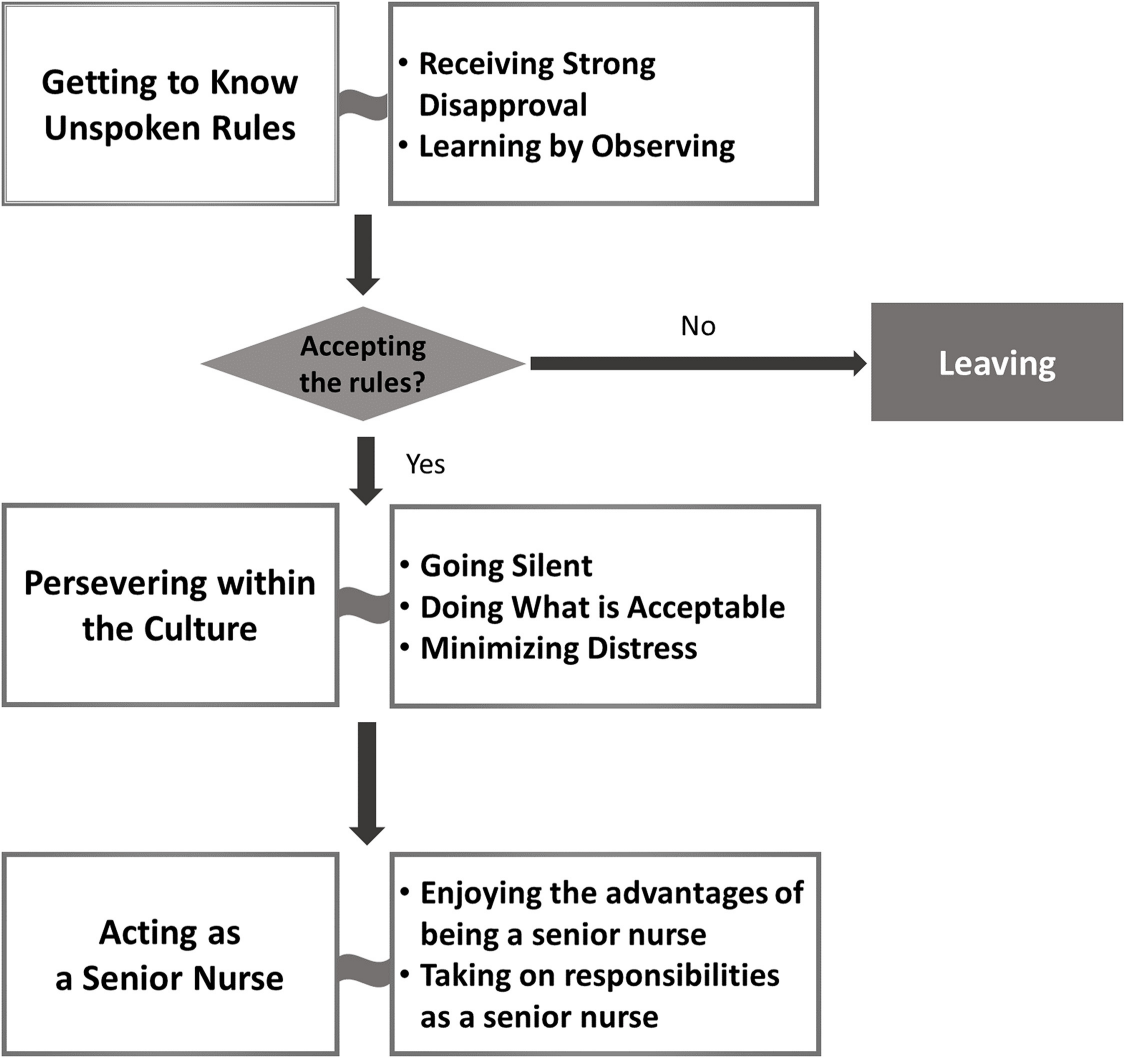
Process of Assimilating to the Hierarchical Culture.

The main social process that explained intra-organizational communication in nurses was “assimilating to the hierarchical culture,” which involved three major phases. Those phases were defined by major goals that the nurses strove to achieve, including getting to know unspoken rules, persevering within the culture, and acting as senior nurse (Fig 1). To acclimatize to the cultures of their assigned wards, nurses learned to be silent and engaged in their assimilation into the established hierarchy. This process explained how the pattern of communication in nursing organizations affected the way in which nurses were assimilated into organizational culture from individual nurses’ perspectives.

### Phase I: Getting to Know Unspoken Rules

The first phase of assimilation, during which nurses became familiar with hospital work and were simultaneously asked to quickly adapt into the culture of their new ward, was the time during which nurses began to learn the unspoken rules of their new environment. In addition to the ways in which specific clinical duties should be performed, unspoken rules of behavior between nurses constituted an important part of ward culture. From this perspective, the major goal for newly hired nurses to achieve in this phase was to learn the unspoken rules as well as what was unacceptable; new nurses learned about unacceptable communication and behavioral patterns as a member of the specific unit. During this phase, nurses learned to discern unacceptable behavior by receiving strong disapproval directly from seasoned nurses or observing the experiences of peers or those of lower rank with little work experience.

#### Receiving strong disapproval

Nurses found that determining the atmosphere of an assigned ward was the most difficult aspect of transitioning from nursing student to new nurse. Learning the unspoken rules of behavior was particularly challenging for members of a ward because new nurses were required to abide by these rules regardless of their personal thoughts or behavioral patterns. Objections to or behaviors deviating from these rules were not tolerated, and there was a tendency to label new nurses as strange if they breached these boundaries.

In some ways, I think the culture here is that whenever a new nurse comes, many nurses try to stick that nurse into a certain category. It’s like, we do it this way, so you should do it this way. (…) She’s supposed to react this way, but when she reacts differently, then she becomes a weird person.

(ID04, p. 5, 8 years of experience)

Nurses consider ready-established standards to be very important. If you do certain things in a way different from such standards or cannot reach those levels, then people would get more and more angry within the shift-base working system. People don’t want to work with you and start hating you, then…what should I say? It’s like you will get in so much trouble that you can’t get away with. I think that’s how it is.

(ID09, p. 3, 8 years of clinical experience)

Although the rules were enforced for new nurses, the moods or work styles of seasoned nurses with whom they worked could affect how strictly such rules were applied to each circumstance, leading to stressful situations for new nurses. Because hierarchy based on length of work experience was the most fundamental mechanism determining how the rules of behavior were applied, the rules could also be perceived as advantageous only to those with experience. The most definitive example of this was the shift report, in which the level of psychological pressure involved varied depending on the personal tendencies of seasoned nurses to whom the work was being handed over.

If an older senior’s turn is after mine, then what she expects of me… That person, when handing over to me, doesn’t have this and that done, but when she works after me, then I have to have everything done. When I see something like that, with no consistency, then I say to myself, “This isn’t right,” but I cannot express it out loud. That’s what a hierarchy is. Anyway, that person is a superior, with seniority over me in the ward, and I rank lower, so I can’t openly say, “That is not right. Why do you demand that from me when you don’t do it yourself?” If I did that, I’d be ostracized. In the ward (laughing), it would lead to something like ‘Who do you think you are?’ being said, so I don’t say anything more. Always, this is in a hierarchical relationship. Should I say, it’s slightly, really authoritative? I have to obey unilaterally; I have to follow whatever was said. Whatever wasn’t done, I have to finish things before leaving. It’s a fact that because of these things, I had to work overtime a bit more. Actually, nurses…I think any ward you go to, they have some kind of a hierarchy. Things like these, well, as long as you remain here [in the hospital], the nurses of lower rank do not say a word and it makes it tough on them.”

(ID08, p. 4, 5 years of clinical experience)

Nurses’ responses to psychological pressure and stressful situations as shown above could vary according to an individual nurse’s personality and interpersonal relationship skills. Their responses to such stressful situations can be classified into two groups. One group of response is “accepting the rules,” characterized by internalizing stress and refraining from expressing opinions; the other is “resisting,” which describes those who actively express their oppositions, different opinions, or needs to seniors or managers.

It was virtually impossible for nurses at the lowest level of ward hierarchy to cope with or resolve situations without risking being reprimanded or attacked verbally; therefore, to avoid conflict, they did not outwardly express their opinions or discuss injustices that they may have suffered, choosing in most cases to survive by accepting the rules. When the relational distress became unbearable, some nurses ultimately chose to leave the relationship by taking a hiatus or resigning. The decision to leave appeared to occur relatively early in their careers, generally within the first two years.

#### Learning by observing

Learning about what is unacceptable does not occur only through one’s own experience. The experience of observing extreme disapproval toward peers served to reinforce the silence and obedience of the nurses to the unspoken rules. Engaging in conflict with senior nurses could lead to even stronger and more frequent disapproval. If such negative experiences were repeated, the working relationship between the new and senior nurses involved worsened; in extreme cases, the problem escalated to the extent that a new nurse was ostracized by the entire ward. By observing these situations, nurses learned to maintain their silence, even if they believed something to be unfair.

There are a couple of peers who would talk back to when our seniors say. Actually she wasn’t talking back. She was just stating what she thought was right. That’s being very brave. But, if you do that, you’ll get buried alive. You’ll be a loner. Because things like that exist, so we can’t say anything.

(ID03, p. 4, 4 years of clinical experience)

The nurses who participated in the study experienced severe psychological stress for 2–3 years after assignment to a specific ward and complained of stress and difficulties due to accommodating senior nurses’ personal working styles, attitudes toward training new nurses, and emotional states, in addition to the need to master ward duties. During this process, communication between nurses was perceived to be mostly unidirectional and to show a tendency of laying blame and focusing on mistakes rather than protecting and supporting new nurses.

I mean, when a problem occurs, we should take the position that we should protect and help her since she is still new and doesn’t know about certain things. But still, it’s gotten better now, but nurse groups tend to say “It’s your fault” and push the person into the corner—at least that’s what I think. I guess that’s why there’s no improvement, and opinions…opinions of new nurses, I mean instead of accepting the opinions of new people, there’s still a strong tendency to quash them. Like, there’s more of ‘Who are you?’ It’s gotten better than before. I think placing importance on hierarchy for even trivial matters might be the reason why more improvements haven’t been made.

(ID06, p. 7, 5 years of clinical experience)

Experienced nurses who were newly hired to a ward were likely to face greater difficulty relative to that experienced by new nurses during this period. Since existing ward members assumed or expected that those with previous experience “should know this much,” veteran nurses received insufficient training and support, and found themselves in situations that they did not fully understand, creating additional stress. When a nurse started working at a hospital that had a different cultural climate from that of her previous workplace, she often found the new work environment more rigid; long-term members remained steadfast in their working procedures and rules and did not welcome any changes. This type of situation also discouraged new members from expressing requests for change or discussing efficiency.

So if you have experience from other hospitals, people simply assume that you should know this much and be able to handle everything because you have that much experience. And don’t give you enough training as well because of that. Newly graduated nurses can receive systemically planned trainings all set in place, but for experienced nurses, they give you a rough instruction for the system—that is all! You will never know the system [electronic medical record system] in detail with such little information. So you have to learn by yourself while you work. Also the severity of patients’ illness here is much higher than my previous workplace. Such things were really hard for me. Some people can adapt and get over it right away but some others can’t…lots of nurses quit their jobs because of it, especially during their first year.

(ID09, p. 7, 8 years of clinical experience)

### Phase II: Persevering within the Culture

During the phase II period, nurses focused their efforts to persevere in the assigned ward by complying with the unspoken rules that were learned via trial and error in the first phase. Because any show of resistance during the first phase resulted in negative outcomes, nurses in this phase worked at smoothly merging into their work unit rather than standing out in any way. Strategies for persevering included going silent, doing what is acceptable, and minimizing distress.

As nurses had more experience, they were able to see conflictive situations or unfavorable working conditions from a broader perspective and tried to make sense of the causes of such circumstances. However, being now in the middle of the hierarchy meant they still had to abide by the rules, especially when working with those of higher rank. Also, issues that arose within the shift-based work system and on-going nursing shortage remained unsolved, which inculcated hierarchical emphasis and thereby strengthened the disadvantages for nurses of lower ranks.

#### Going silent

If nurses were continuously ignored or rejected when voicing their opinions on repetitive unfair situations or poor working conditions, they developed distrust of the nursing manager and entire nursing unit. With the accumulation of such negative experiences, nurses chose to focus on their own survival and ignore any problems or need for change, which reinforced their silence and gradually weakened their will to find ways to express their opinions or improve their work efficiency or the organizational culture.

Well, I talked about some issues, this and that to the nurse manager. I did that several times. But she just listened, literally “listened” and didn’t do anything. After experiencing such things over and over again, then I’m like “Why would I bother to do worthless things?” Rather I need to focus on just getting used to whatever I supposed to do. (laughing) Just gaze on survival here.

(ID11, p. 10, 12 years of clinical experiences)

Further, the stress evoked by a ward’s rules and the chronic nursing shortage within the organizations continued during this phase; under such conditions, those of lower ranks were likely to refrain from claiming their rights.

If you are a nurse and when you get hurt, you [are concerned about] the shift schedule first, before [worrying] about how much you are hurt. Of course your peers worry about you as well, but they also worry about the schedule first. Because nursing work is [a] shift-base system, if I can’t make it, someone else has to fill in. Actually, I’m getting married, but for the wedding, I have to feel bad about the schedule first. That is the thing. More than being congratulated by someone, things are like that. If I’m gone, someone has to work. You can’t just be happy or sad for someone else’s occasions. It’s one of the tough parts.

(ID06, p. 15, 5 years of clinical experience)

#### Doing what is acceptable

While the first phase involved learning about what was unacceptable, the second phase represented the stage at which nurses learned how to express their needs in a manner acceptable to other members of the group. As nurses accumulated experience, they figured out how and when to speak safely and when to maintain their silence. Nurses were likely to feel more comfortable with clearly expressing their opinions regarding work-related issues (e.g., lack of supplies), both formally and informally, to the head nurse or senior nurse(s) on the same shift. Nurses also adopted alternative strategies to voice their opinions, including gathering the opinions of the majority regarding work-related complaints or changes needed and having senior nurses with authority act as intermediaries to express their concerns.

However, such alternative communication paths limited the ability to deliver diverse opinions or discuss sensitive matters, and because nurses feared presenting their opinions publicly as individuals with respect to matters that opposed existing rules or work policies, they tended to become even more silent regarding areas that required change unless a manager or veteran nurse took the initiative.

I do mention it, but changes don’t come easily. “We don’t do it this way here, but it was done in that way in my previous hospital. I think it would be better if we do it this way.” But things didn’t change easily. The superiors…they don’t seem to accept it very well. If what we do is meaningless and isn’t any good for patients, then it should not be done anymore. We all know that, but not a single person would take the lead (for change)…. They all do it, but inside, they all think, “I don’t think this is right.”

(ID03, p. 7, 4 years of clinical experience)

Because nurses chose not to express their opinions under these circumstances, the ward manager’s leadership style was an important factor in facilitating work-related communication. When the leadership or working styles of head nurses welcomed change and actively accepted new ideas, junior nurses were more likely to speak up frequently and actively.

It depends ward by ward. In one ward, you suggest something to change out loud but nothing changes, but in the ward I’m in now, the head nurse listens to us a lot. When we say something, and we can see some changes, even [if] it’s very little.

(ID05, p. 4, 7 years of clinical experience)

She’s [the previous nurse manager] like…how should I say it? It didn’t feel like she was higher than me in a hierarchical relationship, but more like a peer. I said whatever’s on my mind and she was quite receptive, but now, of course I know this is her (the current head nurse) first time being a head nurse but… For example, the duties came out and we expressed our concerns that the member make up was poor—then she just said “that’s how you learn,” and we were just dumped in poor working situations.

(ID02, p. 2, 11 years of clinical experience)

The problem that nurses found most difficult to resolve involved maintaining personal relationships with peers working in the same ward. In conflictive situations with peers, less-experienced nurses showed a stronger tendency to suppress the resultant stress and avoid attracting attention to the problem.

I feel like most people just suffered such distress and time passed by. For issues at [the] organizational level…just like the improvement of shift report culture mentioned before. If programs like that become available, it should improve or resolve some of the issues a little bit. But in terms of relationships among peers or within hierarchy, particularly about psychological conflicts that you feel, there really is no other way than for the lower rank to hold back. You hold back and later you become senior—that is how things become less difficult.

(ID08, p. 13, 5 years of clinical experience)

The quality of personal relationships was also shown to play a critical role in work performance. This was characteristic of the second phase, in which experience began to accumulate, and being personally close served to alleviate conflict between nurses and even with doctors or employees in other departments. For instance, if a mistake was made, a close relationship with the person involved resulted in lenient consequences and quicker resolution of the issue. In contrast, during this phase, if nurses did not get along with senior nurses but were considered to have outstanding capabilities, their work performance protected them from dismissal, despite identification of a shortcoming in personal relationships.

#### Minimizing distress

Minimizing distress was an important task for nurses during this phase to ensure persistence and survival in an institutional culture that emphasizes hierarchy. The strategies that nurses used most frequently were “redirecting the mind” and “separating personal and work life.” Redirecting the mind involved a process in which nurses tried to understand situations from a distance and find positive meanings. By telling outsiders, such as family members or friends who did not work at the hospital, about what they had experienced, they were able to see their situations from a different angle and remain positive by downward comparison. Separating personal and work life was used to prevent the worsening of stress resulting from a particular situation. While newly hired nurses were likely to release stress by gossiping about a common issue with peers in similar situations, experienced nurses tried to separate their personal lives from work by engaging in hobbies unrelated to their jobs and undertaking trips regularly to distract themselves. Such separation was a protective measure to save themselves from being taken over by interpersonal distress.

Actually, those inside the hospital are all stressed, and that’s why I can’t just release it to these people. I just talk to my friends who are not a nurse, I say, “I don’t know why some people act that way.” and then they ask me, from their perspective, “Oh, is that so? Okay, if that’s the case, then that’s possible. But you know what? It’s way too worse out there.” By talking to other people, I see the things I’ve been through from a different perspective and persuade myself like, “Okay, I am not the one in the worst world.”

(ID04, p. 1, 8 years of clinical experience)

We could have done other things and talked about other things, but we’re too focused on the hospital and couldn’t just let it go. We’re just trapped inside. When my hospital work is finished, I need to shut it off, but it felt like everything was intermingled… If not this, there’s nothing to talk about. So I thought myself, “Do I need to keep distance from them?” So I started set my sights on something else.”

(ID07, p. 18, 7 years of clinical experience)

### Phase III: Acting as Senior Nurse

This was the final phase of assimilation into the hierarchical nursing organization culture, during which nurses held higher rank in the ward hierarchy due to longer tenure. In addition, this was the period during which conflictive situations and consequential stress were reduced because they became senior nurses. Nurses in this phase were enjoying the advantages of being senior nurses as well as taking on senior nurse responsibilities in the hierarchy. Perceived responsibilities of senior nurses were supporting nurses in the lower ranks and representing the nurse’s voice.

#### Enjoying the advantages of being a senior nurse

In their work with junior nurses, senior nurses could benefit from having a higher rank by delegating certain tasks. Nurses in this position could also serve as advocates for those of lower rank by openly giving them a voice concerning selected work-related issues. Benefits for senior nurses include, but are not limited to, enjoying a reduced workload, especially being able to limit overtime after a shift; being prioritized for a favorable work schedule; and receiving relatively more days off. Although they relished the benefits, senior nurses also tried to prevent junior nurses from experiencing the relational problems they had endured as juniors; they attempted to avoid involving emotion during preceptorship and provide more support for new nurses at work.

Now as I became a seasoned nurse, I think to myself, ‘I had a hard time because of my senior nurses, so I should not have my juniors go through the same thing’, but once I became a senior… Firstly I get physically tired a lot and my thoughts like ‘I had to do this and that but I will do things differently … Gradually, I started handing over things one by one to the nurses below my rank, like “hey, have this done on my behalf this time”…I guess this is how things repeat themselves over time. I feel frustrated that I am doing the same thing as my senior colleagues did, and it’s hard for me that I don’t make any changes at all. And probably this is how my juniors would feel the same way as I used to.

(ID08, p. 14, 5 years of clinical experience)

‘The boss’ of our ward—that’s how we call her. She doesn’t have any official title though. How should I put this? Things change when she speaks up. She would say things [to the head nurse] like, “Why do I have to take on the disadvantages [more than the junior nurses]?” For example, when we have the work schedule announced and if more night shifts are assigned to her than us, she would fix it by saying, “Why do we have to take so many night shifts?” and say, “When I was at your point in career, I took night shifts very often, but why don’t you do as much?”

(ID03, p. 17, 4 years of clinical experience)

#### Taking on responsibilities as a senior nurse

As their careers continued, participants felt heavy responsibilities in the senior nurse role. Officially as well as unofficially, they needed to train and supervise new nurses and those of less experience. Additionally, if a new nurse made a mistake or error it was often considered not only the individual’s fault, but also likely to be considered the preceptor’s co-responsibility or caused at least in part by her insufficient training. Thus, supporting those nurses in the lower ranks was perceived as a heavy burden for senior nurses in work environments with chronic nursing shortages.

Another important task for senior nurses was representing the nurse’s voice in conflictive situations and in discussions with nurse managers or other department representatives. If there was an issue to discuss and resolve at the ward level, senior nurses would take the lead and voice the staff nurses’ opinions to nurse managers; they become intermediaries, just as those whose help they had received when they were in the middle of the hierarchy during the second phase. Senior nurses endeavored to protect inexperienced nurses by speaking on their behalf in conflictive situations involving nurses in other wards or care workers in other departments.

When we had enough members and [an]other ward needed help, we sent there our junior nurses. They were treated unfairly there. They were so upset, so I told about that to our head nurse. Then she called the head nurse of the ward and said, “If you’re going to be this way, we won’t send any more helpers to your ward.”

(ID02, p. 6, 11 years of clinical experience)

Whatever the case may be, since I’m in this ward, I think taking care of our own members is the right thing to do. I believe that anything unfair that’s thrown our way, we have to deal with it and protect nurses in this ward. I believe that since I am in charge, I have to be the shield when working with my juniors.

(ID15, p. 2, 10 years of experience)

However, the personal efforts of these nurses seldom led to the development of an official support system that extended to the entire ward or nursing organization. With a chronic shortage of nurses and a work environment that provoked mental exhaustion, it was difficult to maintain effort or improvement beyond this level. Although nurses believed that the organizational culture in clinical settings was undergoing gradual improvement, they found that the desire to make a difference that they had had during their early nursing years gradually weakened. At some moment, they realized that they were unwittingly following the same path of many of their senior nurses. They found themselves assimilated into the hierarchy-based organizational culture and acting in the ways their senior nurses had in the past.

Now as I became a seasoned nurse, I think to myself, ‘I had a hard time because of my senior nurses, so I should not have my juniors go through the same thing’, but once I became a senior… Firstly I get physically tired a lot and my thoughts like ‘I had to do this and that but I will do things differently … Gradually, I started handing over things one by one to the nurses below my rank, like “hey, have this done on my behalf this time”…I guess this is how things repeat themselves over time. I feel frustrated that I am doing the same thing as my senior colleagues did, and it’s hard for me that I don’t make any changes at all. And probably this is how my juniors would feel the same way as I used to.

(ID8, p. 4, 5 years of clinical experience)

From the second phase onward, experienced nurses occupied higher ranks in the power hierarchy of a ward; the number of occasions for direct interaction with nurse managers also increased along with such movement in the hierarchy. Still, regardless of their relationships with their nurse managers, the senior nurses still remained of lower rank. From this perspective, they tended to communicate only selected issues to managers, and they were expected to unequivocally accept top-down messages from higher organizational levels, which were often delivered without explanation.

Since our head nurse is a very authoritarian person… I have a much longer career than my juniors, which put me in a position to represent them, so I have to talk with the head nurse a lot. In most cases, however, she ignores what I say, or does not let me bring things up in the first place. She has a strong style of her own… If things don’t fit her way, she would say, “Think before you talk,” or make us do things over and over again until she is satisfied, so sometimes we would do things without even knowing why.

(ID14, p. 18, 19 years of clinical experience)

## Discussion

The results of this study illustrated how the hierarchy-based culture in nursing units was applied and reinforced via communication between organization members in Korea. The results also demonstrated that a culture that emphasizes organizational rigidity, exclusivity, and hierarchy serves as a serious obstacle to active communication within an organization and as a mechanism that can lead to difficult transitions for nurses.

The time spent in each phase varied according to personal characteristics, working conditions, and assigned workplaces. Moreover, each phase was significantly influenced by interactions of the individual nurses with their peers in the same ward, the group culture in assigned units, and the ward managers’ leadership styles. In addition, the characteristics of nursing work—the continuity of patient care needed throughout all shifts—served as an overarching condition reinforcing the members’ silence that was prominent during the assimilation process.

According to the findings, the culture within nursing organizations was perceived as an environment that did not support the transition of new nurses. Adjusting to a new culture was particularly difficult for nurses in transition as the culture contributed to forcing newcomers into almost unconditional obedience during the assimilation process. For these nurses, the first year or so in the assigned ward represented the most difficult period and was the time during which individual nurses began withholding opinions that opposed ward norms. This continued as nurses gained experience, and it transformed into a tendency toward expressing opinions concerning only those issues with which all members of the organization could sympathize.

This phenomenon could be considered consistent with the concept of employee silence, which has been studied as an emerging issue in human resources research during the past decade. Employee silence is defined as “non-communication resulting from a conscious decision of employees to hold back seemingly important information, including suggestions, concerns, or questions” [15]. As nurses experienced the process of assimilation into the hierarchical culture of a nursing organization, they became accustomed to being silent, which further reinforced the silence throughout the process. This phenomenon needs our attention as it has stretched from the individual to the collective level.

Even seasoned nurses showed a strong tendency toward restricting the expression of their opinions to selected issues. Against the background of choosing silence, the strongest influencing factors were fear of the negative consequences of expressing opinions (i.e., perception of risk due to voicing dissent) and the repeated experience of negative outcomes. This phenomenon corresponds with both acquiescent and defensive silence, proposed as types of organizational silence by Van Dyne, Ang, and Botero [16]: silence that occurs at a group level is a significant factor in harming an organization’s productivity and advancement. There is an urgent need to develop intervention programs that break group silence early because collective behavior can be directly linked to patient safety and quality of care in nursing organizations.

The most significant factor in the reinforcement of nurses’ silence was working in a culture in which the nursing organization emphasized hierarchy, an emphasis passed on by senior nurses. Such a culture was perceived to require unconditional acceptance of criticism from senior nurses, difficult working conditions, and the unspoken rules of the assigned ward; it was taken for granted that senior nurses were supposed to have reduced workloads and benefits based on tenure (e.g., numbers of night shifts and days off). However, the degree of emphasis on hierarchy was likely to differ due to individual personalities and working styles, as well as the head nurses’ management styles.

Another significant condition was that nursing care needs to maintain care continuity through shift changes. Shift reporting was a major source of work-related stress for new nurses due to the perception that unfinished work or mistakes from their shift would carry over to the next and accumulate, affecting the image of all nurses or reflecting poor overall work performance. This perception was reinforced within the work culture of hierarchy, which led to situations in which less-experienced nurses were unable to complete their shifts on time and ultimately were forced to work unpaid overtime, either voluntarily or involuntarily.

A chronic nursing shortage was another important influencing factor in the reinforcement of silence. The nursing shortage in Korea in particular has been a chronic problem, as only 60% of all registered nurses are working [17]; even though the number of newly graduated nurses each year has increased, the turnover rate for novice nurses with less than one year of career was about 30–33% [18]. As the participants of this study stated, unless an unexpected absence of a nurse is short (one or two shifts), having substitute personnel from other nursing units or outside of the institution is almost impossible. Thus, such underlying conditions place a continuous burden of training novice nurses on existing nurses without providing alternatives or substitute personnel as needed.

Under such circumstances, catering to an individual’s desires could hold significant consequences for other group members, creating a major psychological burden for nurses. When nurses required leave of any duration because of sickness or injury, they tended to choose to remain at work to reduce the burden on their peers rather than take enough leave and rest; this tendency was emphasized in an unspoken manner and stronger in less experienced nurses. Collectivism, which reflects Korean society’s view that the goals of the group are more important than those of the individual [19], could be a plausible explanation of why such a phenomenon occurs in Korean nurse groups.

Work environments that emphasize hierarchical relationships among nurses, as described above, reflect the Korean cultural impact on communicational behavior within organizations. There have been unfailing attempts to study cultural influence on organizational communication and dimensionalize organizational culture; in this field, Hofstede’s model of cultural dimensions is considered most influential [20]. Beginning with data from a multicultural company’s employees, Hofstede has presented and updated his dimensions to enable cross-national comparison; of the latest six dimensions, three seem to be particularly important to nurses’ communication patterns, namely power distance, individualism/collectivism, and uncertainty avoidance [21].

Hofstede defines power distance as “the extent to which the less powerful members of organizations and institutions accept and expect that power is distributed unequally”. Individualism and collectivism represent opposite ends of a continuum of how strongly and cohesively individuals in a society are integrated into groups, and uncertainty avoidance is based on attitudes toward unstructured situations in a society—comfortable or not ([21], p.9). According to empirical research based on Hofstede’s model, Korea is more hierarchical (score of 60) than the U.S. (40), meaning that people in Korea generally accept an inherent unequal distribution of power within an organization. While the U.S presents extremely individualistic tendencies (score of 91), Korea is collectivistic to a great degree, with a score of 18; a collectivistic society emphasizes loyalty and responsibility to colleague members, and moral terms are likely to be applied to the employer-employee relationship. Compared to the U.S. score of 46, Korea scores 85 for the dimension of uncertainty avoidance, which indicates it is one of the most uncertainty-avoiding countries, with strict rules of beliefs and behaviors that rarely tolerate deviant behaviors or opinions [22].

As illustrated above, the hierarchy-based culture in nursing units can be seen as rooted in national cultural characteristics. Communication patterns in collectivistic societies with high power distance scores, such as Asian countries like Korea, tend to be unidirectional and discouraging of their less powerful members voicing opinions [23]. The findings of this research correspond with such tendencies as the top-down delivery of messages, the spontaneous and forced loyalty to the group at large as well as those with less power to the higher rank in hierarchy in nursing unit, and the rigid implementation of unspoken rules based on the hierarchy with no allowance for deviant thinking or behaviors.

While rigidity and overemphasis of hierarchy exert a strong impact on the promotion and reinforcement of silence, specific ways to resolve conflicts and manage nursing staffs could serve as effective means to break the nurses’ tendency to silence. In the present study, nurses showed a tendency to feel safe about voicing their opinions and speaking freely to those nurse managers who paid close attention to staff experiences or opinions; who were faithful to their own principles of work; and who resolved conflicts without involving personal feelings.

As described above, nurse managers are important agents to effectively control the negative effects of hierarchical culture and create a safe environment for personal opinions. Accordingly, it is necessary for managers to undertake leadership training that focuses on what is needed to enhance communication between members and to create the needed culture for that communication. In addition, systemic support also needs to be provided to maintain and enhance such environments. An interesting finding in the present study was that experienced nurses at the upper level of the ward hierarchy played an important role in the propagation of the organizational culture; it is possible that the Korean organizational culture, which views top-down structure as a prerequisite [24], contributes to such a tendency. Thus, it is also essential to train senior nurses, who hold the role of unofficial intermediary, to build a work environment that encourages horizontal communication.

In considering the hierarchical culture in clinical settings, it is crucial to determine carefully under what circumstances staff nurses are able to voice their opinions. Although there were some minor individual differences, the nurse-participants generally tended to feel more comfortable in private settings, such as one-on-one or small group interactions rather than large-group settings like ward conferences. Moreover, it needs to be noted that, within Asian cultures where power is centralized, expressing opinions via formal involvement of employees is increased only within a strong participatory atmosphere [25]. Thus managers should reinforce upward communication within organizations [26] and encourage members to continue to express their opinions, even if this does not result in change. Managers’ faith in their teams and supports are essential factors in facilitating positive communication [27].

It is also necessary to change the culture supporting deeply-rooted vertical communication between nurses into one of horizontal communication. Horizontal communication refers to communication that occurs between teams, subordinates, and department managers. As peer support is assessed via communication with coworkers or peers before interests are expressed, horizontal communication facilitates personal information sharing [28,29].

In addition, it is necessary to establish a systemic safety net to protect individuals who express opinions that are contrary to those of their peers, superiors, or the organization. Such protection would represent an official organization stance of respecting and valuing the opinions of individual members. More importantly, individual nurses’ perceptions of this support could help form a more advanced organizational culture. Perceived organizational support refers to the degrees of an employee’s belief in “their organization’s commitment to them” (p.500), the belief that the organization values an employee’s commitment and cares about his or her welfare needs [30]. Perceived organizational support has been known to increase organizational commitment and encourage positive work attitudes and behavior [30,31]. The participants in the present study tried to endure problems from one-way communication at the individual level by withholding their opinions, despite the fact that the problems were organization-level issues. Participants also believed that managers and the organization lacked the motivation to intervene. Under such circumstances, assigning all of the responsibility for resolving or managing such issues to the ward manager presents limitations, and a systematic approach to activate horizontal and upward communication is much needed to improve clinical nursing cultures in Korea.

## Conclusion

This study’s results demonstrated that Korean nurses’ intra-organizational communication is a complex process significantly influenced by various elements, such as the overall culture of the nursing organization and the interactions between individual nurses, their peers, and their managers. The study further demonstrated that multiple conditions are involved in maintaining the hierarchical culture and reinforcing organizational silence in nurse groups, including the characteristics of nursing work based on shifts and continuity, the work culture in individual nursing organizations, the leadership characteristics of managers, and the Korean sociocultural environment grounded in collectivism. In order to improve the rigid working atmosphere and culture in nursing organizations and increase members’ satisfaction with work and quality of life, managers and staff nurses require effective communication training within horizontal relationships. Moreover, organization-level protection should be provided to create an environment that encourages free expression.

## Supporting Information

S1 File Initial Interview Guidelines. (DOCX)

S2 File Revised Interview Guidelines. (DOCX)
