# Supplementary material for: Assimilating to Hierarchical Culture: A Grounded Theory Study on Communication among Clinical Nurses
Source: PLoS One. 2016 Jun 2;11(6):e0156305. doi: 10.1371/journal.pone.0156305 (PMC4890802; doi:10.1371/journal.pone.0156305)
Supplement: S1 File — (DOCX) [file pone.0156305.s001.docx]

**Initial Interview Guidelines**

Opening questions

1. Could you please tell me how you are doing at your work these days?

2. Tell me how communication within your workplace is going.

3. Could you tell me what are the difficulties regarding communication among your coworkers?

Additional questions (probes) if topics of interest have not been discussed

1. The participant’s career history:

-“Have you ever worked somewhere else before? For example, a different department, different ward or ICU, or other hospital(s)? Could you tell me about that in a little more detail?

2. If yes to probe#1, try this probe to open up more stories:

- Please tell me about any differences between workplaces.

3. Ask the participant to elaborate each difficulty she or he mentions.

**초기 면담가이드**

**도입 질문**

1. 요즘 병원에서 일하기 어떠신지 말씀해주시겠어요?

2. 일하고 계신 병동에서 의사소통이 어떻게 이루어지는지 말씀해주세요.

3. 일하고 계신 병동의 동료 간호사들과의 의사소통 시 어떤 어려움이 있는지 말씀해주시겠어요?

**다음 주제들에 대해 참여자가 언급하지 않는 경우 부가 질문**

1. 참여자의 임상경력 관련 정보

--현 근무지 외에 다른 곳, 예를 들어 다른 부서, 다른 병동이나 중환자실 혹은 다른 병원 등에서도 일하셨었나요?

--(위 질문에 그렇다고 한다면) 거기에 대해서 좀 더 자세하게 말씀해주시겠어요?

2. 1번 질문에 대한 답이 ‘그렇다’라면 다음 질문들을 이용해서 더 이야기를 들어본다:

--근무지마다 간호사들 간 의사소통에 어떤 차이가 있었는지 말씀해주세요.

3. 참여자가 언급하는 어려움들 각각에 대해서 좀 더 자세히 얘기해보도록 요청한다.
