# Supplementary material for: Assimilating to Hierarchical Culture: A Grounded Theory Study on Communication among Clinical Nurses
Source: PLoS One. 2016 Jun 2;11(6):e0156305. doi: 10.1371/journal.pone.0156305 (PMC4890802; doi:10.1371/journal.pone.0156305)
Supplement: S2 File — (DOCX) [file pone.0156305.s002.docx]

# Revised Interview Guidelines

Opening questions (not revised):

1. Could you please tell me how you are doing at your work these days?

2. Tell me how communication within your workplace is going.

3. Could you tell me what are the difficulties regarding communication among your coworkers?

Additional questions (probes) if topics of interest have not been discussed (revised):

1. Could you tell me about communication challenges you’ve been facing with your superior?

--expand this to “with your more experienced colleagues,” “with less experienced colleagues,” “with other departments or nurses in other units.”

--Could you tell me about when communication in your ward goes well and when it does not? Could you talk about what made the difference(s), please?

2. Other nurses talked about the association between personal relationships among members and work in their working units. Could you tell me about that?

3. How have you changed, if you can think of any, as your career has progressed (or as your rank has moved up over time in your ward)? Could you tell me about that?

--If not brought up: “Other participants talked about changes in their roles as their careers progressed. Could you tell me about that as well?”

4. How do you deal with stress from your work or your coworkers? What works best for you?

5. How do you deal with conflictive situations while working? Have you experienced any? Could you elaborate that please?

**면담가이드(수정본)**

**도입 질문(변경없음)**

1. 요즘 병원에서 일하기 어떠신지 말씀해주시겠어요?

2. 일하고 계신 병동에서 의사소통이 어떻게 이루어지는지 말씀해주세요.

3. 일하고 계신 병동의 동료 간호사들과의 의사소통 시 어떤 어려움이 있는지 말씀해주시겠어요?

**다음 주제들에 대해 참여자가 언급하지 않는 경우 부가 질문(수정됨)**

1. 상급자하고 의사소통할 때 어떤 어려움들을 경험하고 계신지 말씀해 주시겠어요?

--“연차 높은 간호사들”, “신규/연차 낮은 간호사들”, “다른 병동이나 다른 부서 사람들”로 확대시켜서 질문해 볼 것

--언제 소통이 잘 되고, 언제 잘 안 된다고 생각하시는지 말씀해주시겠어요? 무엇 때문에 그런 차이가 생긴다고 생각하는지 말씀해주시겠어요?

2. 다른 분들은 동료 간호사들과의 인간관계 및 그 관계가 업무에 미치는 영향에 대해서도 말씀해주셨는데요, 그 부분에 대해서 말씀해주시겠어요?

3. 경력이 쌓여가면서 (혹은 연차가 올라가면서) 혹시 바뀌었다고 생각되는 부분이 있다면 어떻게 바뀌었는지 말씀해주시겠어요?

--(참여자가 언급하지 않는다면) 다른 분들은 경력이 쌓이면서 본인의 역할이 변해가는 것에 대해 말씀해 주셨는데요, 거기에 대해서 말씀해 주시겠어요?

4. 병동 업무나 동료들 때문에 받는 스트레스는 어떻게 해소하세요? 가장 효과가 있는 것은 어떤 방법인가요?

5. 일하다가 갈등 상황이 생기면 어떻게 대처하세요? 갈등 상황을 경험하신 적이 있나요? 거기에 대해서 자세히 말씀해주시겠어요?
